# Beyond DPA: An Atomistic Framework for a Quantitative Description of Radiation Damage in YBa_2_Cu_3_O_7_

**DOI:** 10.1002/smsc.70303

**Published:** 2026-05-19

**Authors:** Federico Ledda, Daniele Torsello, Davide Gambino, Flyura Djurabekova, Fabio Calzavara, Niccolò Di Eugenio, Ville Jantunen, Antonio Trotta, Erik Gallo, Kai Nordlund, Francesco Laviano

**Affiliations:** ^1^ Department of Applied Science and Technology Politecnico di Torino Turin Italy; ^2^ Istituto Nazionale di Fisica Nucleare Sezione di Torino Turin Italy; ^3^ Department of Physics University of Helsinki Helsinki Finland; ^4^ Department of Physics, Chemistry and Biology Linköping University Linköping Sweden; ^5^ Culham Campus United Kingdom Atomic Energy Authority Abingdon Oxfordshire UK; ^6^ Eni S.p.A. Rome Italy

**Keywords:** binary collision approximation, dpa, HTS, molecular dynamics, radiation damage

## Abstract

Radiation damage in high‐temperature cuprate superconductors represents one of the main technological challenges for their deployment in harsh environments, such as fusion reactors and accelerator facilities.Their complex crystal structure makes modeling irradiation effects in this class of materials a particularly demanding task, for which existing damage models remain inadequate.In this work, we develop an atomistic‐based approach for describing primary radiation damage in YBa_2_Cu_3_O_7_, by coupling molecular dynamics and binary collision approximation simulations in a way that makes them complementary. When integrated with primary knock‐on atom spectra obtained from Monte Carlo codes, our results establish a framework for multiscale modeling of radiation damage, enabling quantitative estimates of several damage descriptors, such as defect production, defect clustering, and the effective damaged volume for any specific irradiation conditions where collision cascades dominate. This computational approach is suitable for the prediction of irradiation effects in any complex functional oxide, with applications ranging from aerospace to nuclear fusion and high‐energy physics.

## Graphical abstract

To quantify primary radiation damage and describe defect morphology in the high‐temperature superconductor YBa_2_Cu_3_O_7_, an atomistic multiscale framework is proposed. Coupling Molecular Dynamics and Binary Collision Approximation simulations and integrating the atomistic description with Primary Knock‐on Atom spectra from Monte Carlo and transport simulations, it yields irradiation‐specific descriptors (defect yield, cluster density, and damaged volume) beyond the conventional displacement‐per‐atom metric (NRT‐dpa), enabling physically grounded predictions for fusion reactors, accelerators, and aerospace applications.

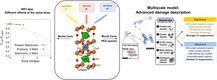

## 1 Introduction

High‐temperature superconductors (HTS), particularly the cuprate YBa_2_Cu_3_O_7−*δ*_ (YBCO), have emerged as leading candidates for efficient high‐field magnets, as their technological maturity now enables operation far beyond the intrinsic field and temperature limits of conventional low‐temperature superconductors [1, 2, 3, 4]. These advances have established YBCO as a key enabling material for compact fusion reactors and next‐generation high‐energy accelerators, where superconductors must operate under intense radiation and extreme environmental conditions, bringing the radiation tolerance of such materials under the spotlight [5, 6, 7]. In harsh radiation environments, energetic particles inevitably interact with the crystal lattice, producing a broad variety of defects that can profoundly alter the functional properties of the material, like the critical current density, *J*_c_ [8], and the superconducting critical temperature, *T*_c_ [9]. Understanding how ionizing radiation affects YBCO is therefore crucial not only to predict its operational reliability but also to unravel the microscopic mechanisms that link defect formation to changes in superconducting behavior [10]. To this end, irradiation studies with different particles, from electrons to light ions and fission neutrons, are being performed to emulate the complex damage environments expected in operation [11, 12, 13, 14, 15].

**FIGURE 1:**
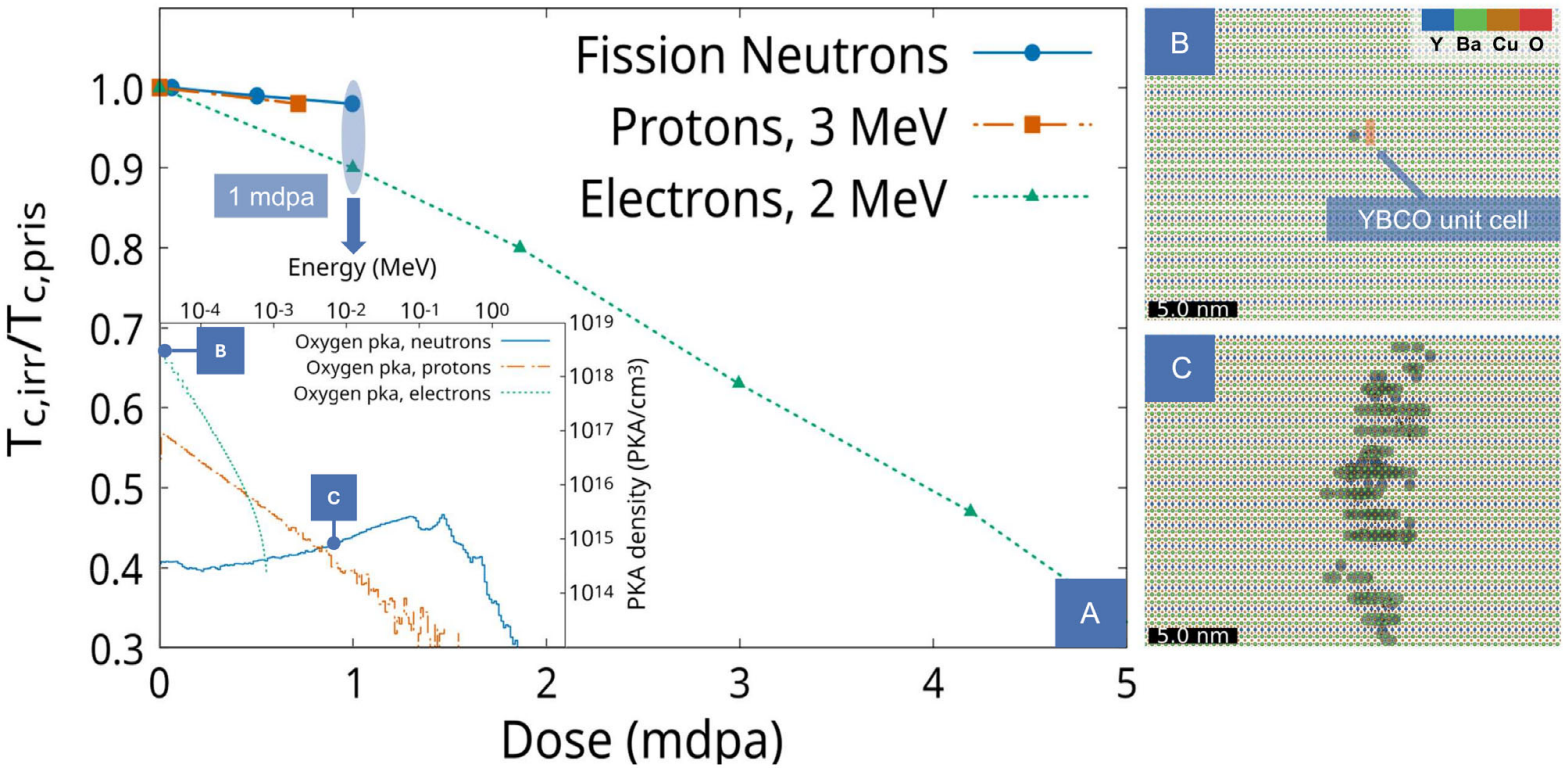
(A) Normalized *T*_c_ of YBCO single crystals under three different irradiation conditions (protons [20], electrons [15], and fission neutrons [21]) plotted as a function of the dose (dpa). For the same dpa, markedly different reductions in *T*_c_ are observed, indicating that dpa alone is insufficient to capture the extent of functionality degradation. The dpa values are estimated by the authors from the original fluence data and experiment descriptions. Oxygen primary knock‐on atom spectra calculated for YBCO at a dose of 1 mdpa are presented in the inset, for electron (dotted line) and neutron (solid line) irradiation. Low‐energy recoils (few eV) dominate under electron irradiation, whereas neutron irradiation produces high‐energy recoils capable of generating extended defect structures. Proton irradiation represents an intermediate regime: The spectrum peaks at low energies, mirroring the divergence of the Rutherford cross section at low scattering angles, but extends well into the keV range owing to the larger proton mass, resulting in a damage morphology intermediate between those of electrons and neutrons. (B) Example of defects produced by a 25 eV oxygen recoil at 20 K, obtained from MD simulations. Interstitial atoms are highlighted by a light‐blue mesh. (C) Example of a large defect cluster produced by a 7 keV oxygen recoil at 20 K, as obtained from MD simulations. Interstitial atoms are highlighted by a light‐blue mesh.

However, meaningful comparison between such experiments requires a common metric of radiation exposure: Particle fluence alone is insufficient, as charge, mass, and energy determine distinct damage mechanisms. Traditionally, this role has been fulfilled by the Norgett–Robinson–Torrens displacement‐per‐atom (NRT‐dpa) concept, widely used to normalize irradiation effects in metallic alloys [16]. Originally developed for monatomic metals on the basis of early simulations, the model offers a fast estimate of damage levels and is routinely implemented in most Monte Carlo (MC) particle‐transport codes. However, it should be regarded primarily as a measure of the energy deposited into the lattice, particularly when applied to complex compounds with mixed covalent and ionic bonding [17]. Several refinements to the original formulation have been proposed in recent years, including thermally activated recombination corrections (CRC‐DPA) and full energy‐range damage models [18, 19]. While these approaches improve the physical description of defect production, they remain largely developed for metallic systems and still reduce radiation damage to a scalar quantity, typically expressed as the number of surviving Frenkel pairs. The limitations of the dpa formulation become evident when the degradation of superconducting properties is examined across irradiation studies employing different particle types. Comparisons among experiments with single crystals [15, 20, 21] employing fully penetrating particles (therefore able to eliminate extrinsic effects such as substrate stress [22, 23]) reveal markedly different reductions in *T*_c_ at comparable dpa values (an example is shown in Figure 1A). The discrepancy is particularly pronounced between electrons and fission neutrons, which differ greatly in charge, mass, and energy transfer per collision. Such divergence, which cannot be reconciled within the dpa formalism, points to the fundamentally different defect landscapes generated by each irradiation type and underscores the need for a more refined description of radiation damage, especially in functional materials.

In the irradiation regimes considered in this work and relevant for most applications of functional materials [24, 25, 26, 27], damage is governed by collision cascades initiated by the first atoms displaced by the incident particles, known as primary knock‐on atoms (PKAs) [28]. These atoms transfer their kinetic energy through successive collisions, generating cascades that ultimately define the defect population and distribution. Because the PKA energy sets the scale and morphology of these cascades, their spectra provide essential insight into the microscopic origin of irradiation effects and can be evaluated computationally, either through MC transport simulations for neutrons [29] or via the McKinley–Feshbach formalism for electrons [30]; computational details for the calculation are provided in the Supporting Information. In YBCO, oxygen PKAs are particularly relevant, as they dominate statistically owing to the low displacement threshold [31]. When their spectra computed at the same dose level (1  mdpa) for the example cases of Figure 1 are compared (inset of Figure 1A), the markedly different recoil‐energy regimes become evident. Under electron irradiation, recoils of only a few eV prevail, with a sharply peaked distribution below the keV range, whereas neutron irradiation produces broad PKA spectra with energies up to several MeV. Proton irradiation occupies an intermediate regime: As charged particles, their spectra are monotonically decreasing with energy and peak in the eV range, mirroring the divergence of the Rutherford cross section at low scattering angles, but the larger proton mass compared to the electron allows significantly larger recoil energies to be transferred, extending the PKA spectrum well into the keV range, where non‐isolated defect production is expected.

Although particle‐transport MC methods provide no direct structural information, the ensuing cascades can be explicitly modeled once the PKA energy is known [6] and a suitable interatomic potential [32] is available for molecular dynamics (MD) simulations. Recoils with energies only slightly above the displacement threshold, which largely dominate under electron irradiation, generate point‐like defects in the form of a few Frenkel pairs, confined within a volume comparable to a single YBCO unit cell (Figure 1B). In contrast, higher‐energy recoils in the keV range, typical of neutrons irradiation, produce extended defect clusters and locally disordered regions spanning tens of unit cells (Figure 1C). The relative abundance of these defect types can explain the different suppression of superconducting properties observed in cuprates, as they introduce scattering centers of different strengths [12, 33, 34] (whose impact does not scale simply with size), yet such distinctions remain inaccessible to existing damage indicators.

Despite their descriptive power, systematic MD simulations of collision cascades are computationally prohibitive and require significant expertise, preventing their routine use for experimental design or for identifying suitable damage proxies across irradiation conditions. A physically informed, multiscale and cuprate‐specific atomistic description of radiation damage is therefore required to bridge the gap between the simplicity of dpa‐based metrics and the accuracy of atomistic simulations. Motivated by these findings, we propose an approach that integrates an atomistic representation of defect formation into MC transport codes, providing a qualitative yet physically grounded description of the damage induced in YBCO.

## 2 Results and Discussion

### 2.1 Method Overview

Constructing an atomistic description of radiation damage in YBCO requires, in practical terms, quantifying the response of the material to PKAs across the full energy spectrum relevant to irradiation (extending from few eV up to several MeV) and establishing a transferable dataset of analyzed cascades spanning the entire PKA energy range. This dataset provides the atomistic foundation for coupling with MC particle‐transport calculations to predict radiation damage under arbitrary conditions in which atomic displacements and collision cascades dominate, while retaining a computational cost and complexity comparable to the NRT model.

MD simulations offer a complete description of collision cascades, capturing many‐body interactions and defect recombination, but their computational cost restricts the applicability of the method to relatively low recoil energies and small simulation volumes [35, 36]. At the opposite extreme, binary collision approximation (BCA) methods efficiently describe the high‐energy ballistic phase of cascades, where atomic collisions are essentially binary and collective effects are negligible [36]. The separation between BCA and MD runs is also well motivated by that cascades induced by ions or recoils with energies exceeding the keV range are split into spatially separated subcascades [37]. Combining these two techniques therefore provides the most efficient route to achieve atomistic coverage over the entire energy spectrum [38, 39, 40].

On this basis, we introduce a hierarchical coupling strategy in which MD and BCA play complementary roles determined by the recoil energy. Cascades initiated by low‐energy PKAs are resolved entirely through MD, capturing the full sequence of atomic rearrangements and defect recombination. For higher‐energy PKAs, the cascade is first evolved with BCA; as the energy of each generated recoil falls below a threshold marking the transition into the many‐body regime, it is treated as the PKA of an independent subcascade and associated with the corresponding MD result previously obtained. The full cascade is then reconstructed by combining the BCA‐generated recoil distribution with the average subcascade properties drawn from the MD dataset, preserving the atomistic accuracy of MD while leveraging the efficiency of BCA.

The MD calculations were performed with the LAMMPS code (compiled for CPU) [41], using a semiempirical potential specifically developed for radiation damage in YBCO [32]. The potential combines a Buckingham form for short‐range interactions with Coulomb terms between charged species; as is standard in radiation damage potentials, the universal Ziegler–Biersack–Littmark (ZBL) potential is smoothly splined at short interatomic separations to capture screened nucleus–nucleus repulsion, and electronic stopping power was introduced as a velocity‐dependent friction term, evaluated with SRIM [42]. The reliability of the adopted potential for radiation damage has been established by the original developers through comparison with DFT, which showed that the predicted defect chemistry is in reasonable agreement with first‐principles results [32]; subsequent independent studies further confirmed this assessment through DFT defect calculations [43] and ab initio MD simulations of threshold displacement energies [44]. The machine learning interatomic potentials currently under development for YBCO promise to further improve the description of atomic interactions in this material in the near future and, once optimized and validated, will be used in combination with the model presented in this work [45, 46].

Collision cascades were simulated for all four PKA species in YBCO (Y, Ba, Cu, O) at 20 and 300 K, with recoil energies logarithmically spaced from 1 eV up to 7 keV. Considering the structural complexity of YBCO, which hosts four crystallographically inequivalent oxygen sites and two copper sites, and the strong directional dependence of the damage at low energies, the sub‐100 eV regime was treated with particular care: 21 initial PKA directions were sampled per crystallographic site, while 11 directions per species were used at higher energies. Defects were identified at 100 ps using the Wigner–Seitz algorithm implemented in the OVITO code [47], counting vacancies, interstitials, and antisites with respect to the perfect reference lattice.

**FIGURE 2:**
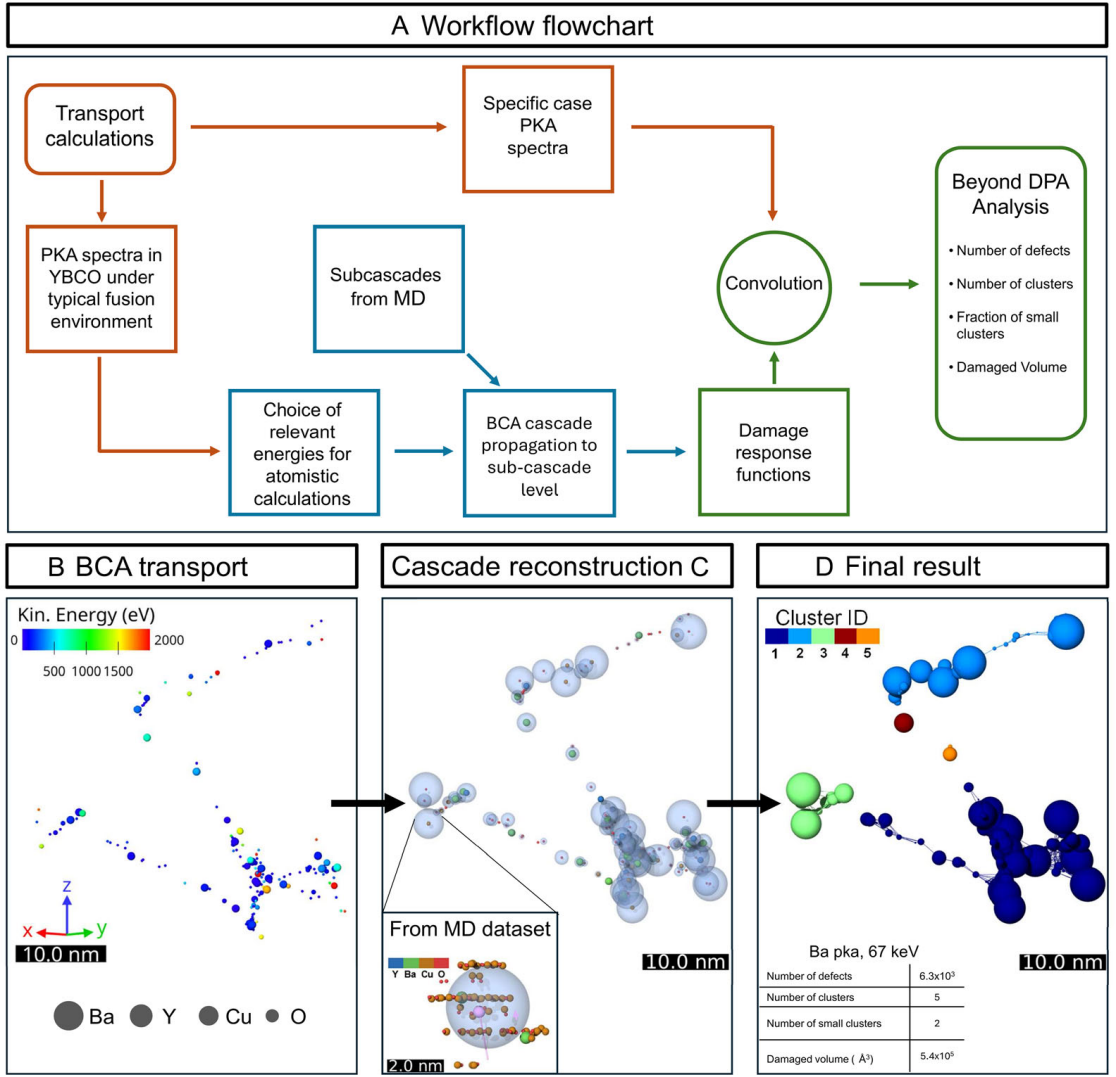
(A) Flowchart of the workflow adopted in this work. (B) BCA propagation of high‐energy cascades: example of recoil distribution from a high‐energy (60 keV) Ba cascade obtained with the BCA code CASWIN [53], illustrating the backbone of large cascades. Each recoil is transported until its energy falls below 2 keV. (C) Cascade reconstruction: Each BCA recoil within the MD energy window (*E* < 2 keV) is replaced by a virtual spherical damage region whose radius *R*_g_(*E*) and defect yield interpolated from the MD dataset. Inset: example of a low‐energy cascade (Ba PKA, 245 eV) from MD simulations, representative of the database of cascades computed up to 2 keV for all atomic species. The defected region is well approximated by a sphere centered along the PKA trajectory with a radius equal to the *R*_g_ of the defect distribution. (D) Final reconstructed cascade and clustering analysis. Clusters are identified by connecting spheres whose surfaces are separated by less than 2*ξ* (*ξ *≈  1 nm), corresponding to the superconducting coherence length in YBCO. The reconstructed geometry can also be exported to CAD format to estimate the total damaged volume using Gmsh [54].

While the total number of produced defects, closely related to the overall disordered fraction, is a useful indicator of irradiation damage, it is insufficient on its own to predict superconducting property degradation in complex functional materials such as YBCO. The arrangement of defects is decisive for superconducting performance: Extended clusters can, in some cases, enhance the *J*_c_ by acting as effective pinning centers [8, 48, 49, 50], whereas small isolated defects predominantly increase charge‐carrier scattering and suppress *T*_c_ and *J*_c_ [12, 51]. Assessing clustering is therefore essential, even though the definition of a “cluster” within a cascade is inherently dependent on the scale considered for the grouping criterion, dictated by the physical properties under investigation. Focusing on the superconducting behavior, we assume that the physically relevant length scale is the low‐temperature Cooper‐pair coherence length, *ξ*(*T* = 0) = 1.12 nm [52], which represents the spatial extent of the paired electronic state. Accordingly, clusters are defined as groups of defects separated by less than 2*ξ* and evaluated using the code OVITO [47]. The resulting distributions of defects are compact throughout this energy range up to 2 keV, and all the low‐energy cascades yielded a single defect cluster under this grouping criterion; the damaged regions can therefore be accurately represented by a sphere centered along the PKA trajectory, with a radius equal to the gyration radius *R*_g_(*E*) of the defect distribution (Figure 2C, inset).

At higher energies, the early ballistic phase of the cascade is modeled using the BCA code CASWIN [53], which transports each recoil through successive binary collisions until all sub‐recoils fall within the energy range covered by the MD dataset, thereby defining the backbone of the large cascades (Figure 2B). Accurate threshold displacement energies *E*_d_ are required as input for the BCA; these were computed with MD using the same interatomic potential adopted for cascades, and their orientation‐averaged values are listed in Table 1.

**TABLE 1:** Threshold displacement energies (in eV) adopted in the BCA simulations.

*T*, K	Y	Ba	Cu1	Cu2	O1	O2	O3	O4
20	35.9	14.8	9.0	27.2	8.7	24.0	24.4	13.5
300	33.2	18.0	9.7	26.7	8.8	22.1	23.5	12.0

Interestingly, *E*_d_ values in YBCO are markedly lower than those typical of metallic alloys or simpler oxides, a characteristic common to layered perovskites in which several lattice sites present shallow potential wells [55]; this is particularly pronounced for oxygen, which is easily displaced by the surrounding heavy cations, and is of particular relevance for the superconducting properties of the material [56, 57]. For each PKA species, 1250 cascades were simulated at 27 energies logarithmically spaced up to 3 MeV at 20 and 300 K, providing adequate statistics across the full high‐energy regime relevant to neutron irradiation.

The high‐energy backbone obtained from BCA is then coupled to the MD dataset to reconstruct the full cascade geometry. Each recoil within the MD energy window is regarded as the PKA of an individual subcascade and replaced by a virtual damage region centered along its trajectory, represented as a sphere whose radius corresponds to the *R*_g_(*E*) derived from MD simulations at the same recoil energy. The defect yield associated with each recoil is likewise interpolated from the MD dataset. As long as the defect yield from individual recoils remains limited, so that the damaged volume is not saturated, the interaction between subcascades can be neglected and their independent treatment provides an accurate approximation. The morphology of the reconstructed cascades is defined by the ensemble of these virtual regions and the associated defect yields (Figure 2C). With the cascade geometry reconstructed, the analysis focuses on the spatial organization and connectivity of the resulting damaged regions (Figure 2D). Since explicit Frenkel pair coordinates are not retained in this representation, the clustering analysis described above is applied to the ensemble of virtual spheres: Two spheres are assigned to the same cluster if the distance between their surfaces is less than 2*ξ*, consistent with the criterion adopted for the MD cascades. This choice is physically motivated: Given the density of the subcascade region, the individual Frenkel pairs produced by a single recoil are spatially confined, and it is therefore reasonable to treat their collective effect on the superconducting condensate as that of a single extended defect region, rather than as a collection of independent point defects. The total volume affected by defects is obtained by computing the volume of each cluster using Gmsh [54], yielding a quantitative measure of the fraction of material structurally perturbed by irradiation. Clusters whose volume is smaller than that of a sphere with radius *ξ* are in this work classified as *small clusters*, representing isolated defect configurations most relevant for charge‐carrier scattering.

The upper boundary of the MD regime, and therefore the transition to BCA, was set at 2 keV. Above the keV range, collision cascades fragment into spatially separated subcascades, whose mutual interactions become negligible [37]; at the same time, all MD cascades below this threshold yield a single compact defect cluster under the grouping criterion described above, allowing each sub‐recoil to be unambiguously associated with a unique spherical damage region in the reconstruction workflow.

**FIGURE 3:**
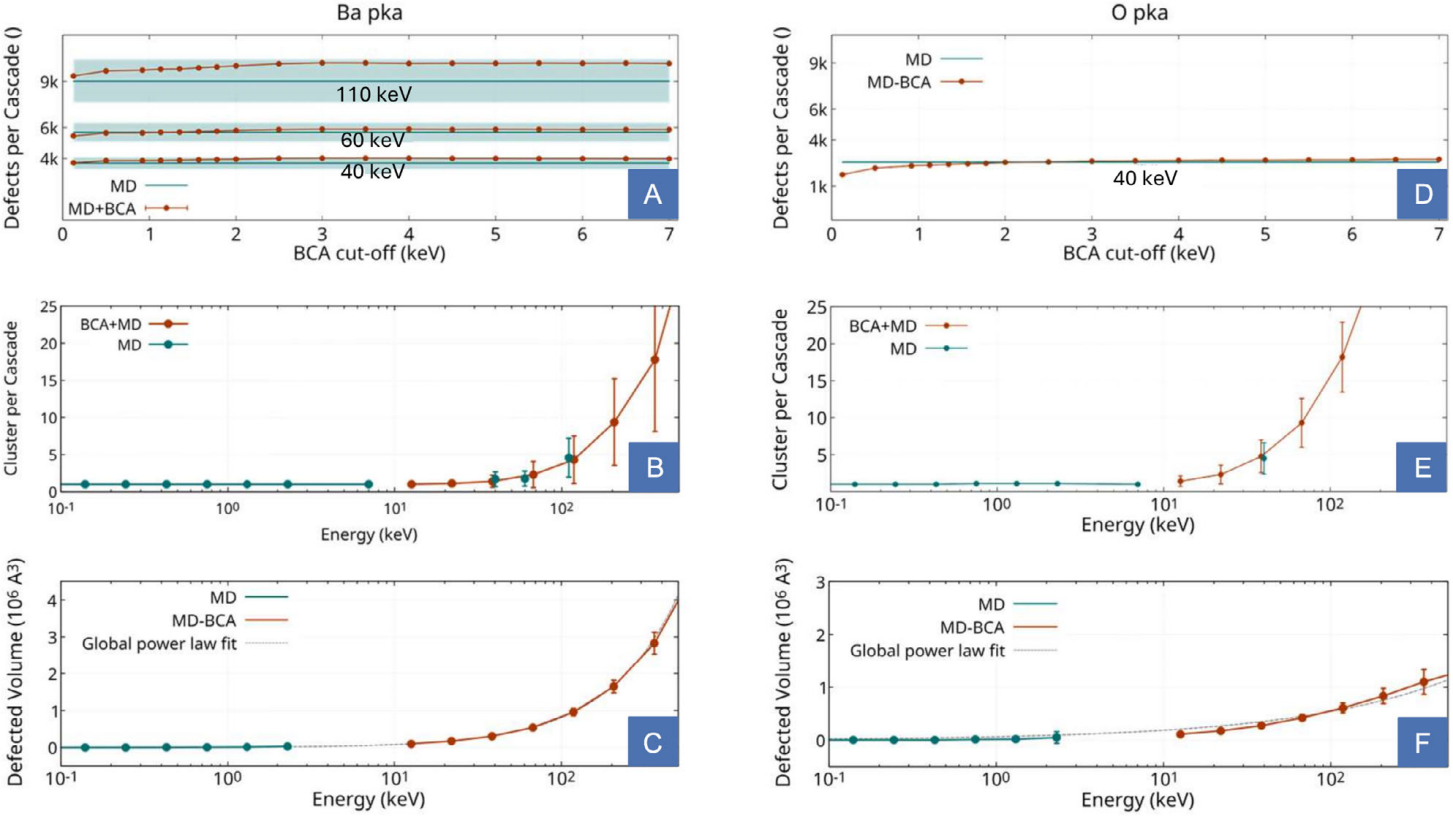
Benchmark of the predicted number of defects per cascade (A,D), clustering (B,E), and damaged volume (C,F) for the heaviest (Ba, left column) and lightest (O, right column) PKA species in YBCO. The proposed model shows excellent agreement with pure MD simulations across all tested quantities. A power law fit over the full dataset demonstrates continuity between the low‐energy, MD‐resolved regime and the high‐energy BCA–MD region.

With this hierarchical coupling approach, excellent agreement is obtained with respect to full MD simulations of large cascades that were performed at 40, 60, and 110 keV for Ba pka and at 40 keV for O (details in the Supporting Information), confirming the validity of the scheme. The combined BCA–MD method reproduces the average defect count predicted by pure MD within its statistical variability for all tested cutoff values and becomes insensitive to the precise choice of threshold above 2 keV, confirming the suitability of the selected transition energy (Figure 3A,D). We note that, since the method relies on an off‐lattice BCA, it does not account for channeling recoils, which produce a fundamentally different damage morphology and fall outside the scope of the present framework. Excellent agreement is also found in the predicted clustering statistics (Figure 3B,E), confirming that the reconstruction preserves not only the total defect yield but also the spatial organization of the damage. A direct comparison of the damaged volumes requires some care. In pure MD, the volume is estimated from the gyration radius *R*_g_ of the defect distribution, an approximation well justified for compact cascades, but increasingly unreliable at higher energies where cascade fragmentation renders *R*_g_ a poor proxy for the total damaged volume. In the BCA–MD reconstruction, on the other hand, the volume is computed directly from the reconstructed geometry using Gmsh [54], properly accounting for the fragmented morphology. Continuity between the two regimes is thus demonstrated by a power law fit over the full dataset (Figure 3C,F): A single function describes the data across the entire energy range, confirming that the BCA–MD volumes connect smoothly with the MD estimates in the regime where the latter remain physically meaningful. We note that for Ba PKAs, the benchmark was performed at three high‐energy reference points with the same statistic adopted for the MD dataset employed in the model (10 directions per energy), whereas for O PKAs, obtaining equivalent statistics proved computationally demanding, owing to the large simulation cells and small timesteps required for light, fast recoils. Nevertheless, excellent agreement between the two models is found across the full range where the comparison is possible.

**FIGURE 4:**
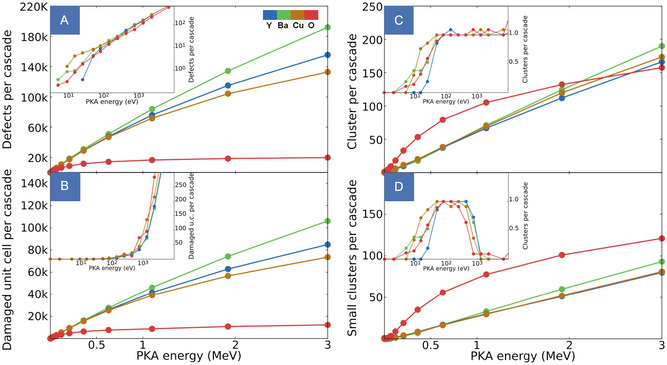
Defect yield (A) and total damaged volume (B) per cascade as a function of PKA energy for each atomic species at 20 K. The sublinear scaling reflects the decreasing efficiency of defect production with increasing cascade size, while heavier PKAs transfer momentum more effectively, generating denser damage. Number of clusters (C) and small clusters (D), defined as defect regions with a volume smaller than that of a sphere of radius *ξ *≈  1 nm per cascade. Y, Ba, and Cu cascades exhibit comparable clustering behavior, whereas O‐induced cascades produce a markedly higher fraction of small, spatially isolated clusters. Insets in all panels highlight the low‐energy regime resolved by direct MD simulations. The non‐monotonic trend observed for the small‐cluster counts originates from a sorting effect: At low energies, nearly all clusters are small, leading to an initial rise; as cascades merge into larger defect regions, the number of small clusters decreases, before increasing again when fragmentation into subcascades appears at higher energies.

The resulting dataset quantifies novel damage descriptors such as the defect yield (Figure 4A), damaged volume (Figure 4B), and clustering statistics (Figure 4C,D) for each PKA species across the full recoil‐energy spectrum. The sublinear increase in defect production with energy reflects the progressive reduction in lattice damage efficiency. The number of atomic displacements produced by a PKA does not scale linearly with its kinetic energy, but rather with the fraction deposited into nuclear collisions, the damage energy *ν*(*E*), the remainder being dissipated through electronic interactions without contributing to lattice disorder [16]. This partitioning is strongly species‐dependent and governs the observed scaling with PKA mass: Heavier atoms maintain a nuclear stopping power comparable to their electronic stopping across the entire energy range studied, so that *ν*(*E*) grows quasi‐linearly with energy, resulting in larger and denser cascades. Oxygen, by contrast, exhibits a characteristic saturation in defect production beyond ≈0.5 MeV: Its nuclear stopping peaks near 10 keV and has already collapsed by 500 keV, so that at higher energies the gain in kinetic energy is almost entirely offset by the decreasing nuclear stopping fraction. This energy partitioning directly determines the plateau in defect yield and damaged volume observed for O (see Supporting Information). Clustering analysis reveals that cascades initiated by Y, Ba, and Cu PKAs share a similar morphology, producing fewer but larger and denser clusters, while oxygen cascades are markedly more fragmented. Beyond the lower total defect count established above, the smaller nuclear stopping cross section of O results in a longer mean free path between successive collisions, producing a spatially dilute damage distribution in which defects rarely agglomerate. As a consequence, O‐induced cascades dominate the small‐cluster statistics across most of the energy range (Figure 4D). Finally, it is worth noting that while the damage descriptors considered in this work provide a comprehensive description of radiation damage in YBCO at the present level of approximation, additional transfer functions can in principle be extracted from the same atomistic dataset and incorporated into the framework, making the model inherently extensible.

### 2.2 Application to Representative Irradiation Cases

Having established the atomistic response of YBCO to individual recoils, the framework can now be applied to realistic irradiation scenarios to quantify the resulting damage landscape. In particular, electron and fission‐neutron irradiations, each corresponding to an accumulated dose of 1 mdpa as discussed in the introduction, provide an ideal benchmark to test the model and to elucidate the distinct degradation behaviors that the NRT‐dpa framework fails to capture.

Although the two irradiation scenarios correspond to identical nominal doses, the defect landscapes obtained convolving the respective PKA spectra with the atomistic response functions differ markedly (as detailed by our results shown in Table 2): The total number of Frenkel pairs is comparable, as expected for equal NRT‐dpa values, yet the model resolves a pronounced divergence in the resulting defect morphology. Electron irradiation, dominated by low‐energy oxygen recoils (inset Figure 1A), produces a high density of small, spatially isolated clusters, each typically containing fewer than 10 defects. Neutron irradiation, by contrast, generates energetic PKAs, leading to extended cascades and dense regions comprising hundreds of Frenkel pairs. These model predictions are fully consistent with well‐established experimental observations and allow a quantitative treatment of the phenomenon, capturing the distinct defect landscapes arising from different irradiation types, providing information far richer than a single dose metric and offering a physically grounded basis for designing and interpreting irradiation experiments.

**TABLE 2:** Predicted radiation damage metrics for YBCO under equivalent nominal doses (1  mdpa). Values are obtained from the coupled MD–BCA dataset convolved with the respective PKA spectra. Despite identical doses, neutron irradiation produces fewer but larger and denser clusters, whereas electron irradiation yields a higher density of small, spatially isolated defects.

Quantity	Neutron irradiation	Electron irradiation
Nominal dose (NRT‐mdpa)	1	1
Frenkel pair density (cm^−3^)	5.48 × 10^20^	2.45 × 10^20^
Cluster density (cm^−3^)	1.44 × 10^18^	3.45 × 10^18^
Small‐cluster fraction (%)	53	99
Mean defects per cluster	405	7
Damaged volume fraction (%)	0.98	1.22

## 3 Conclusion

In conclusion, this work demonstrates the intrinsic limitations of simple dose‐based metrics such as the NRT‐dpa when applied to complex functional materials, and introduces a multiscale computational framework that couples MD with the BCA to provide a physically grounded description of radiation damage, using YBCO as a case study. The approach overcomes the intrinsic restrictions of pure MD, limited to the keV energy range and small supercells, by leveraging BCA to access the high‐energy regime. The resulting MD–BCA dataset establishes a transferable link between atomistic physics and engineering‐scale predictions, offering detailed structural insight at a computational cost comparable to that of routine MC calculations and far beyond the descriptive power of standard NRT‐dpa models. When applied to realistic irradiation conditions, the framework captures the distinct defect morphologies induced by different particle types, enabling quantitative characterization of the damaged landscape through complementary metrics such as *defect yield*, *cluster density*, and *damaged volume fraction*. This capability provides a powerful interpretative tool for existing experiments and a predictive basis for the design of future irradiation studies. While the present framework offers a detailed atomistic picture of radiation‐induced disorder, establishing direct links between these structural changes and the evolution of superconducting properties remains an open challenge. Progress in this direction will rely on expanding the experimental basis, particularly through systematic studies on single crystals under well‐controlled irradiation conditions, together with continued theoretical efforts aimed at connecting the microscopic defect landscape to macroscopic functional behavior. In parallel, extending the computational analysis to longer timescales will be essential to capture defect migration, recovery processes, and the long‐term evolution of the irradiated microstructure.

## Supporting Information

Additional supporting information can be found online in the Supporting Information section.

## Funding

This work was supported by Research Council of Finland (349690), EUROfusion (101052200), and Ministero degli Affari Esteri e della Cooperazione Internazionale (US23GR16).

## Conflicts of Interest

The authors declare no conflicts of interest.

## Supporting information

Supplementary Material

## Data Availability

The data that support the findings of this study are available from the corresponding author upon reasonable request.
