# Supplementary material for: Beyond DPA: An Atomistic Framework for a Quantitative Description of Radiation Damage in YBa2Cu3O7
Source: Small Sci. 2026 May 19;6(5):e70303. doi: 10.1002/smsc.70303 (PMC13189514; doi:10.1002/smsc.70303)
Supplement: Supplementary file 1 — Supplementary Material [file SMSC-6-e70303-s001.pdf]

Supplementary Material for:
Beyond dpa: an atomistic framework for a quantitative description of radiation damage in $YBa_2Cu_3O_7$

CONTENTS

In this Supplementary Material we provide the technical background and information necessary to reproduce the calculations and analyses presented in the main text, together with additional figures and physical interpretation supporting the discussion of the results. We report the full set of simulation parameters used in the MD calculations up to 2 keV, the BCA settings adopted to model the high-energy ballistic phase, and the MD-BCA coupling scheme employed to construct a unified description of collision cascades across the entire PKA-energy range. Details on the reconstruction algorithm, clustering analysis, and validation against full atomistic simulations are also included, together with additional figures and tables supporting the conclusions of the study.

1 | DETAILS OF THE MD SIMULATIONS

MD simulations constitute the foundational component of this work. They provide a direct representation of collision cascades for PKAs with energies up to 2 keV and, through coupling with the BCA, supply detailed information on the damage produced at higher energies.
All MD calculations were performed with the LAMMPS code (compiled for CPU)[1], using a semiempirical potential expressly developed for radiation damage in YBCO[2]. The potential combines a Buckingham form for short-range interactions with Coulomb terms between charged species; as is standard in radiation-damage potentials, the universal Ziegler–Biersack–Littmark (ZBL) potential is smoothly splined at short interatomic separations to capture screened nucleus–nucleus repulsion. Electronic stopping power was introduced in the calculations as a friction term, and it was evaluted with the code SRIM[3].

Collision cascades were simulated for PKAs of the four atomic species in YBCO (Y, Ba, Cu, O) at 20 K and 300 K, with energies logarithmically spaced from 1 eV to 7 keV, as presented in Tab. 1. Because the initial PKA direction has a stronger impact at lower energies, and given the structural complexity of YBCO, with four crystallographic O sites and two Cu sites, the sub-100 eV regime was treated with particular care. For initial energies smaller than 100 eV, we sampled 21 random initial directions for each crystallographic site; for higher energies, 11 initial directions per atomic species were used. For the MD–BCA coupling, only cascades with energies $\leq$ 2 keV were retained, as this marks the transition between the many-body regime and the ballistic regime. The justification for this cutoff is provided in Section 3.

The simulation cell size was chosen according to the cascade energy (details in Tab. 1). For each cascade, a dedicated supercell was independently minimized with the *quickmin* routine and then equilibrated for 40 ps in the NPT ensemble, avoiding the use of a common pre-equilibrated configuration across runs. This ensures uncorrelated starting states and removes any dependence on a particular initial cell. During the cascade stage, a central, spherical, core region was defined to evolve in NVE for 100 ps without external perturbations, while on a surrounding boundary region a velocity-rescaling thermostat was imposed, to absorb heat and momentum.
For Ba PKAs at 20 K, additional high-energy cascades at 40, 60, and 110 keV were performed to provide reference data for benchmarking the BCA-MD reconstructed cascades. For O PKAs, a reference set of cascades at 40 keV was also computed; further extending the benchmark was not feasible within the available computational resources. Ten initial directions were tested. To reduce the computational cost associated with the large system size, the equilibrated starting cells were taken from a single long equilibration run, writing restart files every 5 ps; each cascade was then initialized from a different restart to maintain independence while lowering the equilibration overhead.

T (K)	E_k PKA (eV)	Supercell size (replicas)
20, 300	1, 2, 5, 9, 15, 25, 50, 80	$30 \times 30 \times 10$
20, 300	140, 245, 430, 750	$45 \times 45 \times 15$
20, 300	1310, 2300, 4050	$60 \times 60 \times 20$
20, 300	7000	$60 \times 60 \times 20$
20	$40 \times 10^3, 60 \times 10^3, 110 \times 10^3$ *(Ba PKA only)*	$480 \times 480 \times 160$
20	40×10^3 *(O PKA only)*	$480 \times 480 \times 160$

TABLE 1 Y, Ba, Cu, O pkas, list and details of the performed simulations

For the aims of this work, different analyses were performed on the MD results using the OVITO code[4]. In particular, a Wigner–Seitz analysis was employed to compute the number of defects produced by each cascade, considering vacancies, interstitials, and antisites. The numbers reported in the paper for each energy are the averages, over all tested directions, of the defect counts at 100 ps.

Given that the spatial extent of defects impacts superconductivity[5], we performed a cluster analysis to quantify how point defects are distributed. Using OVITO's default modifiers, defects were (i) grouped into clusters by a distance criterion and (ii) characterized by each cluster's radius of gyration R_g. When the defect distribution is compact, as in small cascades, the volume of the affected region is approximated by a sphere of radius R_g. In this scheme, two defects belong to the same cluster if their separation is less than a user-defined cutoff r_c. For YBCO, the relevant length scale is the Cooper-pair coherence length $\xi(T{=}0)\approx 1$nm; accordingly, we set the cutoff to $r_c{=}2$nm. This choice also had the practical advantage that all low-energy cascades yielded a single defect cluster with $R_g \lesssim 2$ nm, a property we exploited in the BCA-based approach.

2 | DETAILS OF THE BCA SIMULATIONS

The off lattice BCA was used to access the higher-energy regime, in combination with MD. All BCA simulations were performed with the in-house code CASWIN[6], developed at The University of Helsinki. To maintain consistency with the MD setup and the experimental conditions of neutron irradiation, all PKAs were launched from the bulk of the material. Accurate threshold displacement energies E_d were required for the calculations; we computed these with LAMMPS[1] for the present work, and a full account will be reported in a separate paper. Direction-resolved E_d maps were generated for all species and the orientation-averaged values adopted in the BCA production runs are listed in Table 2 . For each PKA species, 1250 cascades were simulated in CASWIN to obtain adequate statistics, sampling 27 energies logarithmically spaced up to 3 MeV at 20 K and 300 K. After choosing a transition energy separating the BCA and MD regimes, each recoil was transported in the BCA framework until its kinetic energy dropped below that threshold. The resulting recoils were then stored for further analysis, recording their species (Y, Ba, Cu, O), position (x, y, z), kinetic energy, direction of motion (polar angles θ and azimuthal angle ϕ), and generation label (PKA, SKA, etc.).

T (K)	Y	Ba	Cu1	Cu2	O1	O2	O3	O4
20	35.9	14.8	9.0	27.2	8.7	24.0	24.4	13.5
300	33.2	18.0	9.7	26.7	8.8	22.1	23.5	12.0

TABLE 2 Threshold Displacement Energies (in eV) used in the BCA simulations

3 | CASCADE RECONSTRUCTION PROCEDURE

The main purpose of coupling MD with the BCA was to retain atomistic consistency while accessing higher-energy regimes that are not directly tractable with detailed MD calculations for their computational cost. Specifically, we aimed to obtain (i)

the number of primary defects surviving after cascade cooling (e.g., at 100 ps), (ii) the clustering distribution of the defects, and (iii) an estimate of the volume of material affected.

3.1 | Combining MD and BCA

As discussed in the main text, each recoil generated by CASWIN was treated as an independent PKA giving rise to a smaller sub-cascade. For each recoil, the number of resulting defects was obtained by linearly interpolating the MD dataset as a function of recoil energy, and the total defect count for a reconstructed cascade was computed as the sum over all recoils in the BCA tree. The final number of defects per PKA and energy was obtained by averaging over the 1250 cascades simulated with CASWIN.

To assess the validity of this approach and to determine an appropriate transition energy between the BCA and MD regimes, we compared the predictions of the combined BCA–MD method with full MD simulations performed for Ba PKAs at 40, 60, and 110 keV , and for O at 40 keV at 20 K. Figure 1 shows the total number of defects predicted by the coupled approach as a function of the transition energy, for cutoff values ranging from a few eV up to 7 keV. In all cases, the results remain within the statistical spread of the full-MD data, confirming that the assumptions underlying the reconstruction are sound. In addition, the predicted number of defects become essentially insensitive to the chosen cutoff once the transition energy exceeds about 2 keV.

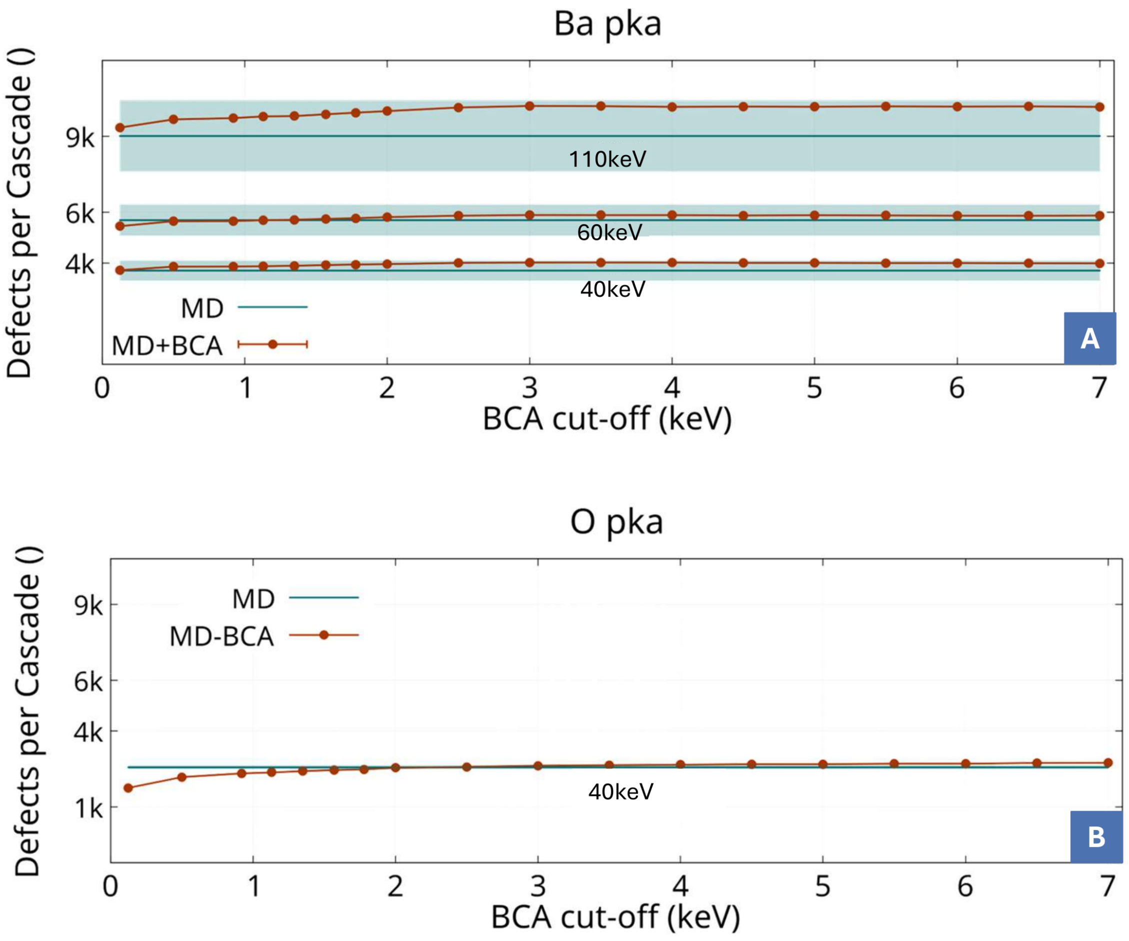

FIGURE 1 Number of defects per PKA predicted by pure MD and by the combined BCA–MD approach, as a function of the transition energy used between the two methods, for Ba and O pka. For all tested cutoff values, the predictions of the coupled method fall within the statistical variability of the full-MD results.

For these reasons, we adopted a cutoff of 2 keV. This choice is also convenient in practice, because all MD cascades below this energy produce a single compact defect cluster under the grouping criterion defined above and detailed later. This, in turn, allows each recoil to be represented by a unique spherical damage region in the reconstruction workflow.

3.2 | Cluster analysis of large cascades from combined MD and BCA

Evaluating clustering in the reconstructed cascade obtained combining MD with BCA is slightly more involved than in pure MD. With OVITO it is possible to perform cluster analysis on a defect distribution, where defects belong to the same cluster if their separation is below a user-defined cutoff. Since the superconducting coherence length $\xi \approx 1-2$ nm[7] sets the relevant spatial scale in YBCO, we adopted r_c=2ξ, i.e., r_c =2.24 nm.

In reconstructed cascades, however, explicit defect coordinates are not available, because the number of defects is obtained by interpolating from recoil energy. We therefore used an MD-informed geometric proxy, exploiting the fact that the defect clouds produced by recoils in the MD range are compact and well approximated by a sphere centered along the PKA direction of motion, with radius equal to the radius of gyration R_g of the MD defect distribution (such a radius being defined as the root mean square distance of defects from the cluster center of mass). Analyzing the MD dataset, it is clear that once the threshold displacement energy is exceeded, cascades initiated by PKAs with energies below 2 keV generally produce a single defect cluster (Fig. 2), with occasional deviations attributable to reasonable statistical noise.

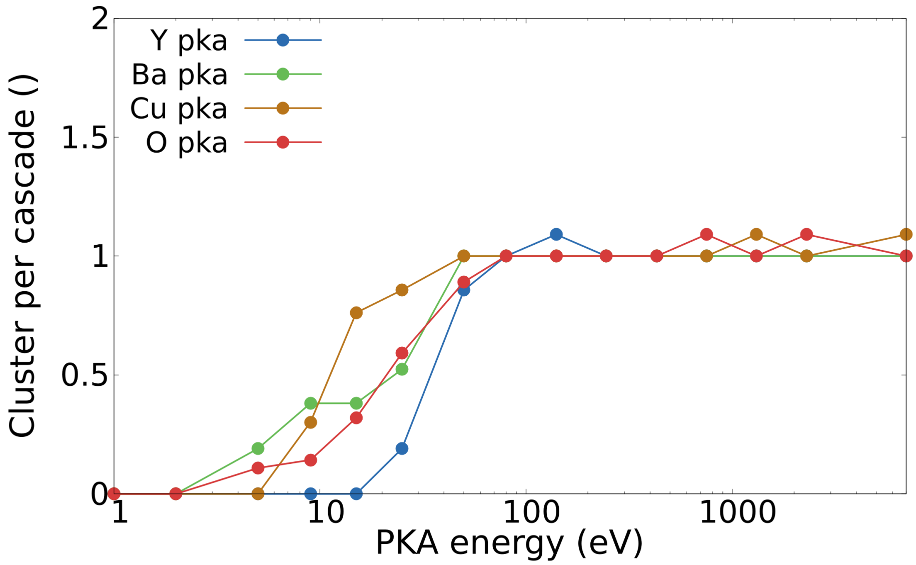

FIGURE 2 Average number of defect clusters per cascade in the MD dataset for PKA energies below 2 keV at 20 K, computed using the clustering criterion defined in the text. All cascades in this energy range yield at most one compact cluster, apart from statistical fluctuations, a property that greatly simplifies the geometric reconstruction of larger cascades within the coupled BCA–MD framework.

Building on the previous observations, each CASWIN recoil was replaced by a virtual spherical particle whose radius R_g was interpolated from the MD dataset as a function of PKA species and energy, and whose center was placed along the PKA direction at an offset equal to R_g from the recorded recoil position. Because standard cluster analysis of OVITO ignores particle radii (it uses center–center distances), we instead constructed an explicit bond network: two spheres were connected if the distance between their surfaces was less than the chosen clustering cutoff r_c (i.e. if $d_{i,j} - (R_i + R_j) <= r_c$). We then applied OVITO's bond-based cluster analysis to this graph.

Once the cascades were reconstructed with the virtual particles, we estimated the volume of material affected by defects by exporting the reconstructed geometry to a CAD format and computing volumes with Gmsh[8]. The frequency of small clusters

(defined as those with a volume smaller than that of a sphere of radius ξ) was then evaluated from these cluster volumes. High-energy Ba cascades from MD were also used as a reference for the cluster analysis of the reconstructed cascades. The agreement between MD and MD+BCA is excellent, as shown in Fig.3 of the main manuscript.

3.3 | Energy partitioning and PKA species dependence of damage descriptors

The damage descriptors presented in Fig 3A–D of the main text exhibit a non-trivial dependence on PKA species and energy, which warrants a more detailed physical interpretation than can be accommodated in the main text. In particular, the saturation of defect yield and damaged volume for O PKAs, and the markedly different clustering statistics of light versus heavy species, originate from the strongly species-dependent partitioning of the PKA kinetic energy between nuclear and electronic stopping channels. To support this interpretation, we provide here the relevant stopping power curves and damage energy functions, computed for all four PKA species in YBCO.

The number of atomic displacements produced by a PKA, indeed, does not scale linearly with its kinetic energy, but rather with the fraction deposited into nuclear collisions , the damage energy $\nu(E)=\eta(E)\cdot E$, the remainder being dissipated through electronic interactions without contributing to lattice disorder[9]. This partitioning is strongly species-dependent and governs the observed scaling of all damage descriptors with PKA mass. To illustrate this, we computed the nuclear stopping power for all four ionic species in YBCO using the ZBL formulation, approximating the target by its compositional averages Z_2=22.6 and M_2=51.2, and compared it with the electronic stopping power as evaluated by SRIM[3] and employed in the simulations (Fig. 3).

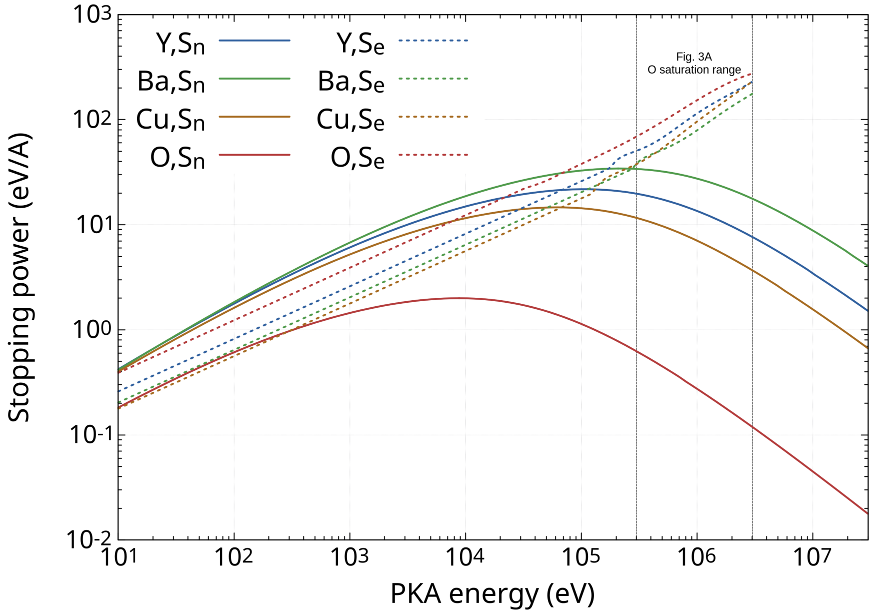

FIGURE 3 Nuclear and electronic stopping powers for the four PKA species in YBCO (Y, Ba, Cu, O) as a function of PKA energy. The nuclear stopping power was computed using the ZBL formulation with compositional averages Z_2=22.6 and M_2=51.2; the electronic stopping power was evaluated with SRIM. The collapse of the nuclear stopping of O above 500 keV contrasts sharply with the behaviour of the heavier species, for which nuclear and electronic contributions remain comparable across the full energy range.

The difference between O and the heavier species is immediately apparent. The nuclear stopping of O peaks near 10 keV and has already collapsed by 500 keV, so that above this energy electronic interactions dominate the energy loss entirely. By contrast, the nuclear stopping powers of Y, Ba, and Cu peak at much higher energies and remain comparable to their respective electronic stopping powers across the full range studied. As a consequence, the damage energy $\nu(E)$ for O saturates around 10^5 eV and remains nearly constant thereafter, while for Y, Ba, and Cu it continues to grow quasi-linearly (Fig.4),maintaining a difference of

approximately one order of magnitude with respect to O at 3 MeV.The saturation in defect yield and damaged volume observed for O in Figs. 3A-B of the main text is therefore a direct and quantitative consequence of this energy partitioning.

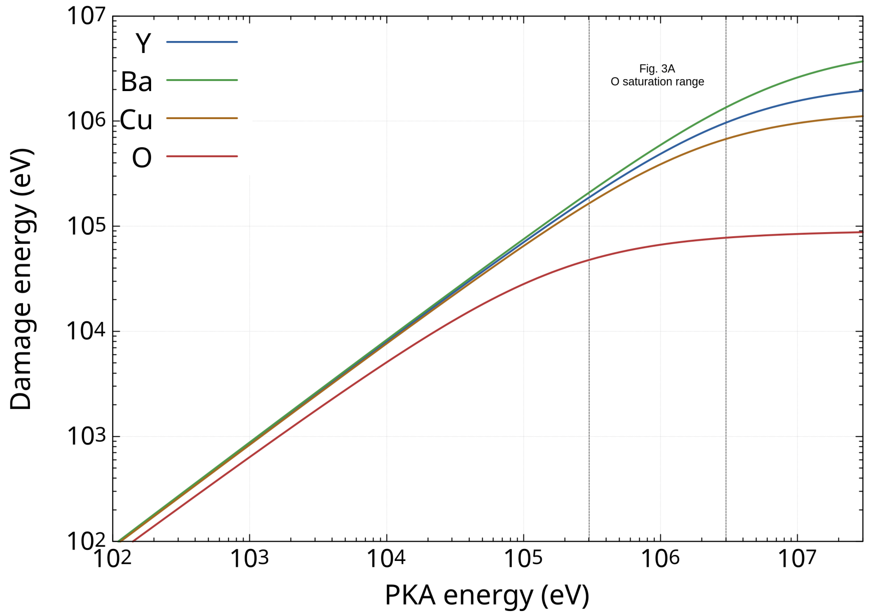

FIGURE 4 Damage energy $\nu(E)$ as a function of PKA energy for the four species in YBCO, computed using the Lindhard partitioning function as parametrised by Norgett et al.[9], assuming average target properties. The saturation of $\nu(E)$ for O above 10^5 eV, in contrast to the quasi-linear growth of the heavier species, directly accounts for the plateau in defect yield and damaged volume observed in Figs. 3A-B of the main text.

Regarding the cluster statistics (Fig. 3C-D), the interpretation requires additional consideration. The number of clusters depends not only on the total defect count, but primarily on their spatial distribution within the cascade. For O PKAs, two competing effects are at play: the lower total defect count established above, and the considerably longer mean free path between successive nuclear collisions, a direct consequence of the smaller nuclear stopping cross section, which results in a spatially dilute cascade in which defects are produced further apart. Because successive defects are frequently separated by distances exceeding the grouping distance each defect or small group tends to form an independent cluster rather than agglomerating. O therefore produces a higher density of small, spatially isolated clusters despite its lower total defect count, whereas heavier PKAs, whose denser cascades promote agglomeration and thermal spike formation, produce fewer but larger clusters.

4 | MONTE CARLO SIMULATIONS

MC simulations of neutron irradiations were carried out with the PHITS code[10,11] with the native nuclear data library JENDL-4.0 in the ACE format[12], the Event Generator Mode[13] and Intel compiled MPI parallelization on the ENI-HPC6[14]. The models of the irradiated samples were developed by the authors directly in PHITS, in constructive solid geometry. The simulation consisted of a planar neutron source implementing the spectrum measured in the experimental channel of the TRIGA reactor, emitting collimated neutrons toward a pure YBCO crystal (simulated density: 6.3 g/cm^3), with a statistics of 4.7 x 10^9 Monte Carlo particles.

PKA spectra from protons were obtained via the BCA code TRIM[3], simulating 3 MeV protons incident on a 30 µm thick pure YBCO crystal (simulated density: 6.3 g/cm^3, with a statistics of 10^6 Monte Carlo particles, using the Detailed Calculation with Full Damage Cascades mode. TRIM does not provide directly the PKA spectra, but it keeps track of the recoils initiating each subcascade after being displaced by the incident particles, which matches the definition of PKA. It was therefore possible to compute the spectra with an ad-hoc post-processing script; the spectra were computed on the same multigroup structure adopted

in the neutronic calculation, including 200 energy bins.
The PKA spectra from electron irradiation were obtained with an in-house implementation based on the first-order McKinley–Feshbach correction to the Mott cross section for relativistic Coulomb scattering [15]. The differential cross section for producing a recoil atom of energy T was implemented following the formulation of Lucasson [16]:

$$\frac{d\sigma}{dT} = CZ^2 \frac{1-\beta^2}{\beta^4} \frac{T_m}{T^2} \left[1 - \beta^2 \frac{T}{T_m} + \pi\alpha\beta \left(\sqrt{\frac{T}{T_m}} - \frac{T}{T_m}\right)\right] \tag{1}$$

where the maximum kinematically allowed energy transfer is:

$$T_m = \frac{2EM\left(E + 2m_e c^2\right)}{\left(Mc^2 + m_e c^2\right)^2 + 2EM} \tag{2}$$

Here Z is the atomic number of the target atom, M its mass, m_e the electron mass, and E the kinetic energy of the incoming electron. The variable T denotes the transferred energy to the recoil atom, while T_m is the maximum allowed transfer given by Eq. 2. The parameter $\beta = v/c$ is the relativistic velocity ratio of the electron, and α is the fine-structure constant. The constant $C \simeq 2.5 \times 10^{-29}$ m^2 collects the fundamental constants entering the Lucasson expression in SI units.

The resulting differential cross sections were then integrated above the displacement threshold and convolved with the incident electron fluence to generate the PKA spectra, taking into account the stoichiometry and density of YBCO. To obtain the atomistic predictions, the PKA spectra relative to the different irradiation scenarios were convolved with the dataset generated by the MD–BCA approach using a Python script with linear interpolation, similar to the procedure used in [17].

REFERENCES

1. Thompson AP, Aktulga HM, Berger R, et al. LAMMPS - a flexible simulation tool for particle-based materials modeling at the atomic, meso, and continuum scales. *Comp. Phys. Comm.*. 2022;271:108171. doi: 10.1016/j.cpc.2021.108171
2. Gray RL, Rushton MJD, Murphy ST. Molecular dynamics simulations of radiation damage in YBa2Cu3O7. *Superconductor Science and Technology.* 2022;35(3):035010. doi: 10.1088/1361-6668/ac47dc
3. Ziegler JF, Ziegler MD, Biersack JP. SRIM – The Stopping and Range of Ions in Matter. *Nuclear Instruments and Methods in Physics Research Section B.* 2010;268(11-12):1818–1823. doi: 10.1016/j.nimb.2010.02.091
4. Stukowski A. Visualization and analysis of atomistic simulation data with OVITO-the Open Visualization Tool. *MODELLING AND SIMULATION IN MATERIALS SCIENCE AND ENGINEERING.* 2010;18(1). doi: 10.1088/0965-0393/18/1/015012
5. Unterrainer R, Gambino D, Semper F, et al. Responsibility of small defects for the low radiation tolerance of coated conductors. *Superconductor Science and Technology.* 2024;37(10):105008. doi: 10.1088/1361-6668/ad70db
6. Pugacheva TS, others . *Nuclear Instruments and Methods in Physics Research Section B: Beam Interactions with Materials and Atoms.* 1998;141:99–104.
7. Gauzzi A, Jönsson-Akerman BJ, Clerc-Dubois A, Pavuna D. Scaling between superconducting critical temperature and structural coherence length in $YBa_2Cu_3O_{6.9}$ films. *Europhysics Letters.* 2000;51(4):667–673. doi: 10.1209/epl/i2000-00390-9
8. Geuzaine C, Remacle JF. Gmsh: A three-dimensional finite element mesh generator with built-in pre- and post-processing facilities. *International Journal for Numerical Methods in Engineering.* 2009;79(11):1309–1331. doi: 10.1002/nme.2579
9. Norgett M, Robinson M, Torrens I. A proposed method of calculating displacement dose rates. *Nuclear Engineering and Design.* 1975;33(1):50-54. doi: https://doi.org/10.1016/0029-5493(75)90035-7
10. Sato T, Iwamoto Y, Hashimoto S, et al. Recent improvements of the particle and heavy ion transport code system – PHITS version 3.33. *Journal of Nuclear Science and Technology.* 2024;61(1):127–135. doi: 10.1080/00223131.2023.2275736
11. Iwamoto Y, Hashimoto S, Sato T, et al. Benchmark study of particle and heavy-ion transport code system using shielding integral benchmark archive and database for accelerator-shielding experiments. *Journal of Nuclear Science and Technology.* 2022;59(5):665–675. doi: 10.1080/00223131.2021.1993372
12. Iwamoto Y, Sato T, Hashimoto S, et al. Benchmark study of the recent version of the PHITS code. *Journal of Nuclear Science and Technology.* 2017;54(5):617-635. doi: 10.1080/00223131.2017.1297742
13. Iwamoto Y, Niita K, Sakamoto Y, Sato T, Matsuda N. Validation of the event generator mode in the PHITS code and its application. In: EDP Sciences. 2007:945–948
14. HPC4. online; 2025. Available at https://www.eni.com/en-IT/media/press-release/2018/01/eni-boots-up-hpc4-and-makes-its-computing-system-the-worlds-most-powerful-in-the-industry.html.
15. Lucasson PG, Walker RM. Production and Recovery of Electron-Induced Radiation Damage in a Number of Metals. *Phys. Rev.*. 1962;127:485–500. doi: 10.1103/PhysRev.127.485
16. Lucasson AM. The Rate of Atomic Displacements Produced in Solids by Electron Bombardment. *Physical Review.* 1962;127(2):485–491. doi: 10.1103/PhysRev.127.485
17. Torsello D, Mino L, Bonino V, et al. Monte Carlo analysis of the oxygen knock-on effects induced by synchrotron x-ray radiation in the $Bi_2Sr_2CaCu_2O_{8+\delta}$ superconductor. *Phys. Rev. Materials.* 2018;2:014801. doi: 10.1103/PhysRevMaterials.2.014801
